# Microsatellite and Mitochondrial Data Provide Evidence for a Single Major Introduction for the Neartic Leafhopper *Scaphoideus titanus* in Europe

**DOI:** 10.1371/journal.pone.0036882

**Published:** 2012-05-18

**Authors:** Daciana Papura, Christian Burban, Maarten van Helden, Xavier Giresse, Benoit Nusillard, Thomas Guillemaud, Carole Kerdelhué

**Affiliations:** 1 Université. Bordeaux, ISVV, UMR 1065 SAVE F-33140 Villenave d’Ornon, France; 2 INRA, ISVV, UMR 1065 SAVE F-33140 Villenave d’Ornon, France; 3 UMR 1202 BIOGECO, INRA Bordeaux, Cestas, France; 4 UMR Ecosystèmes forestiers, CEMAGREF, Domaine des barres 45290, Nogent sur Vernisson, France; 5 UMR 1301 I.B.S.V. (INRA-UNSA-CNRS), BP 167-06903 Sophia Antipolis, France; University of Veterinary Medicine Hanover, Germany

## Abstract

*Scaphoideus titanus,* a leafhopper native to North America and invasive in Europe, is the vector of the Flavescence dorée phytoplasma, the causal agent of the most important form of grapevine yellows in European vineyards. We studied 10 polymorphic microsatellite loci and a 623 bp fragment of the mitochondrial cytochrome oxidase II gene in native *S. titanus* from north-eastern America and introduced European populations, to elucidate the colonization scenario. Consistent with their recent history, invasive European populations were less genetically diverse than American populations for both types of markers, suggesting a recent bottleneck. Significant isolation by distance was detected between American populations but not between European populations. None of the European mitochondrial haplotypes was found in the American vineyards, from which they are assumed to have originated. The precise source of the invasive *S. titanus* populations therefore remains unclear. Nevertheless, the high heterozygosity of North-East American populations (which contained 92% of the observed alleles) suggests that this region is part of the native range of *S. titanus*. Clustering population genetics analyses with microsatellite and mitochondrial data suggested that European populations originated from a single introduction event. Most of the introduced populations clustered with populations from Long Island, the Atlantic Coast winegrowing region in which *Vitis aestivalis* occurs.

## Introduction

International trade has facilitated the movement of species, both in general and for particular taxonomic groups, but only a small fraction of the species transported become established, and only about 1% of the established species become pests [1]. Nevertheless, introduced pest species present a major threat to the environment and cause major economic losses due to of deleterious effects of their spread and the costs of control. The success of these invasive organisms has been attributed to escape from natural enemies, the usurpation of empty niches, an ability to use disturbed habitats and changes in the genetic characteristics of invasive populations [2], [3], [4]. Moreover, worldwide homogenization of the environment due to agriculture has greatly decreased the magnitude of the evolutionary response required to adapt to the conditions in new, geographically distant territories, and this has increased the invasion success of crop pest species [5].

Molecular genetics provides powerful tools for invasion studies, making it possible to link introduced populations to their potential sources, to identify genetic changes associated with shifts between areas and to infer the occurrence of evolutionary processes [6]. Elucidation of the routes by which undesirable organisms are introduced is essential for the development of effective management strategies and sustainable science-based policies [5]. A precise identification of the source population within the native range can also guide the choice of auxiliary agent strains for biological control [7].

The Nearctic leafhopper, *Scaphoideus titanus* Ball (Homoptera: Cicadellidae: Deltocephalinae), is the main vector of Flavescence dorée phytoplasma, a wall-less intracellular bacterium restricted to phloem sieve tubes that is highly pathogenic to several major grapevine cultivars, rapidly leading to the death of the vine [8], [9], [10]. Flavescence dorée is an emerging plant disease in Europe, due to the recent association of a widely cultivated plant (*Vitis vinifera*), a newly introduced insect vector (*S. titanus*) and a local native phytopathogen (the Flavescence dorée phytoplasma) [11], [12], [13].

*S. titanus* was first observed in 1932 by Ball [14], and was described as a common leafhopper with a large range in the United States (particularly in the eastern and central states) and eastern Canada [15], [16], [17], [18]. *S. titanus* was first captured in open woodlands and herbaceous vegetation, but *Vitis* species were subsequently identified as a host plant for this insect [19]. More recently, *S. titanus* has been reported to be limited to a more restricted area, encompassing New York State [20], [21], Virginia [22], Illinois [23] and Canada [24]. Interestingly, catches in these regions were more numerous in woodlands and wild *Vitis riparia* stands than in commercial vineyards [20], [22].

In Europe, *S. titanus* was first reported in 1958, in the vineyards of south-western France [25]. At around the same time, *S. titanus* was also reported in south-eastern France [19], north-western Italy [26] and southern Switzerland [27]. Flavescence dorée was declared a quarantine disease in Europe in 1983 [28], but, despite regulations designed to protect against the disease, including compulsory large-scale insecticide treatments in the first colonized European vineyards, *S. titanus* continued to spread, to Spain in 1996 [29] and Portugal in 1999 [30], western Switzerland 1996 [31] and to Austria in 2004 [32], Serbia in 2004 [33], Croatia in 2005 [34], Hungary in 2006 [35] and Slovenia in 2007 [34]. It is not possible to unravel the complexity of the colonization routes involved from historical data alone. We therefore used population genetics approaches to trace the geographic origin of invaders and to determine the most likely invasion scenarios.

Early efforts to trace the geographic origins of introductions and to investigate the patterns of *S. titanus* colonization and expansion were based on the use of random amplified polymorphic DNA (RAPD) markers. European *S. titanu*s populations were found to be less diverse and structured than American populations. The low level of genetic variability in Europe was interpreted as a consequence of the recent introduction of *S. titanus*, and the lack of genetic structure was considered to result from the transport of grapevine canes, leading to human-mediated dispersal [36]. However, the RAPD technique is subject to a number of limitations, mostly relating to reproducibility, dominance and homoplasy [37]. In this study, we aimed to confirm and extend these preliminary findings, through the use of more appropriate molecular markers, which should be more powerful for the detection of genetic variation and the assignment of individuals to potential source populations. We used microsatellite markers, which are codominant, highly polymorphic and reproducible, together with a mitochondrial marker to track the evolution of the maternal lineage. Microsatellites have high mutation rates and can therefore be used for the small-scale resolution of demographic events [38], [39], whereas mitochondrial DNA (mtDNA) sequences are often used in analyses of earlier phylogeographic events and for analyses of large-scale patterns of genetic diversity [40], [41], [42]. Comparison of the results obtained for microsatellite and mtDNA markers, which have different modes of inheritance and degrees of polymorphism, may provide insight unattainable through studies of either of these types of data alone [43]. Genetic analyses were conducted on *S. titanus* samples collected in north-eastern America from four different *Vitis* spp. (*V. aestivalis*, *V. labrusca, V. vinifera* and *V. riparia*) and in Europe (France, Italy and Switzerland) from *V. vinifera*. We investigated the invasion history of *S. titanus*, by addressing the following questions: 1) Is there any difference in genetic diversity between invasive and native populations? 2) Do nuclear microsatellites and mitochondrial sequences provide concordant information about the invasion scenario? 3) Did European populations originate from a single introduction or multiple introductions from North America?

## Materials and Methods

### Insect Sampling and DNA Extraction

**Figure 1:**
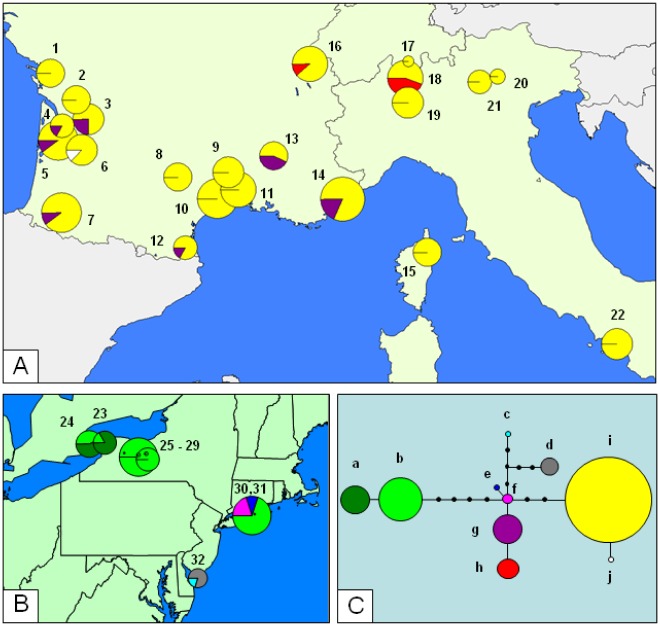
Geographic distribution of *S. titanus* mitochondrial haplotypes in: A Western Europe, B NE America and C the Haplotype network.

We sampled 692 *S. titanus* individuals from 2004 to 2006, at 10 sites in north-eastern America (NE America hereafter) and 22 sites in Western Europe (Table 1 and Figure 1). Individual leafhoppers were sampled from different grapevine plants (no more than one insect sampled per plant with a distance between sampled individual host plants of at least 10 m), with a vacuum insect net collector. Samples collected from the same vine plots during the same period were considered to belong to a single population. In NE America, 10 *S. titanus* populations were collected in 2004 from unmanaged vines growing wild in four viticulture areas: Long Island (Hither Hills-1 and Hither Hills-2) and Outer Coast Plain (Rio Grande) for *V. aestivalis*, Niagara Peninsula (Niagara-1 from *V. labrusca* and Niagara-2) for *V. vinifera* and Finger Lakes (Geneva-1, Geneva-2, Geneva-3, Cornell University and Dresden) for *V. riparia* (Table 1). In Europe, 22 *S. titanus* populations were collected between 2004 and 2005 in France, Switzerland and Italy (the first countries to be colonized), from cultivated *V. vinifera*. In France, 15 populations were collected from the main viticulture areas: Cognac (Boutiers and Lignières-Sonneville), Bordeaux (St Jean de Duras, Margaux, Couhins, Seyches and Monein), Midi-Pyrénées (Connac), Languedoc-Roussillon (Cannes-et-Clairan-Gard, Assas, Mudaison, Trouilles), Provence (Valréas and Antibes) and Corsica (Casamozza) (Table 1). Four populations were collected from Switzerland in 2004: three from the Tessin region (Cugnasco, Castelrotto and Pedrinate), the wine-growing area in the southern Alps in which this leafhopper was first observed in Switzerland [27], and one from the Geneva region (Anierès), the wine-growing area in the northern Alps in which *S. titanus* was observed 30 years later, in 1996 [31] (Table 1). In Italy, two populations were collected in 2005 from Trentino (Novaledo and Arco) and one population was collected in 2006 from the Campania viticulture area in the Naples region (Tramonti) (Table 1).

**Table 1:** Host plants and geographic location of collection sites for *S. titanus.*

Country	Viticulture areas	Population name	Map	Latitude	Longitude	Year of	Host plant	N-10	N- mtDNA
			code			sampling		loci	
France	Cognac	Boutiers	1	46°05′13.73′′N	0°14′28.32′′W	2004	*V. vinifera*	32	7
		Lignères-Sonneville	2	45°33′48.66′′N	0°10′37.86′′W	2004	*V. vinifera*	0	7
	Bordeaux	St. Jean de Duras	3	45°15′14.40′′N	0°51′27.82′′E	2004	*V. vinifera*	0	8
		Margaux	4	45°3′15.22′′N	0°40′19.15′′W	2004	*V. vinifera*	0	6
		Couhins	5	44°45′17.07′′N	0°34′09.85′′W	2004	*V. vinifera*	30	10
		Seyche	6	44°43′12.85′′N	0°18′13.34′′E	2005	*V. vinifera*	12	8
		Monein	7	43°18′39.45''N	0°35′28.99''W	2004	*V. vinifera*	30	10
	Midi-Pyrénées	Connac	8	43°54′54.07′′N	2°33′9.23′′E	2004	*V. vinifera*	0	7
	Languedoc-Roussillon	Cannes et Clainan-Gard	9	43°54′3.54′′N	4° 5′10.29′′E	2004	*V. vinifera*	0	8
		Assas	10	43°42′57.34′′N	3°53′44.82′′E	2004	*V. vinifera*	30	10
		Mudaisson	11	43°37′53.57′′N	4°02′17.39′′E	2004	*V. vinifera*	25	9
		Trouilles	12	42°36′42.33′′N	2°48′46.85′′E	2004	*V. vinifera*	0	6
	Provence	Valreas	13	44°23′10.64′′N	4°59′30.64′′E	2004	*V. vinifera*	30	7
		Antibes	14	43°34′42.42′′N	7°7′28.14′′E	2004	*V. vinifera*	0	11
	Corsica	Casamozza	15	42°31′14.98′′N	9°26′23.43′′E	2004	*V. vinifera*	25	7
Switzerland	Geneva	Anierès	16	46°16′33.85′′N	6°13′25.10′′E	2004	*V. vinifera*	24	9
	Tessin	Cugnasco	17	46°11′5.20′′N	8°55′45.00′′E	2004	*V. vinifera*	0	3
		Castelrotto	18	45°59′34.59′′N	8°50′15.96′′E	2004	*V. vinifera*	24	9
		Pedrinate	19	45°49′34.91′′N	9°0′47.85′′E	2004	*V. vinifera*	24	8
Italy	Trentino	Novaledo	20	46°1′22.73′′N	11°22′0.80′′E	2006	*V. vinifera*	30	4
		Arco	21	45°54′54.99′′N	10°52′31.33′′E	2006	*V. vinifera*	28	6
	Napoli	Tramonti	22	40°41′42.42′′N	14°38′27.38′′E	2005	*V. vinifera*	33	8
USA	Niagara Peninsula	Niagara-1	23	43°09′1.71′′N	79°25′0.56′′W	2004	*V. vinifera*	22	8
		Niagara-2	24	43°08.6.90′N	79°25′2.27′′W	2004	*V. labrusca*	26	8
	Finger Lakes	Geneva-1	25	42°51′9.46′′N	77°20′2.97′′W	2004	*V. riparia*	15	8
		Geneva-2	26	42°51′3.13′′N	77°00′8.67′′W	2004	*V. riparia*	19	4
		Geneva-3	27	42°49′1.09′′N	77°24′3.81′′W	2004	*V. riparia*	30	5
		Cornell University	28	42°50′6.89′′N	77°59′9.14′′W	2004	*V. riparia*	45	5
		Dresden	29	42°40′7.06′′N	76°57′0.59′′W	2004	*V. riparia*	24	4
	Long Island	Hither Hills-1	30	40°57′0.06′′ N	72°39′4.613W	2004	*V. aestivalis*	26	12
		Hither Hills-2	31	40°56′48.61′′N	72°40′45.84′′W	2004	*V. aestivalis*	15	13
	Outer Coast Plain	Rio Grande	32	39°01′0.19′′N	74°53′9.45′′W	2004	*V. aestivalis*	11	6

N-10 loci, number of *S. titanus* individuals genotyped with 10 microsatellite loci; N-mtDNA, number of *S. titanus* individuals sequenced for tRNA^LEU^-COII. For map codes, see Figure 1.

The sampled individuals were immediately placed in 96% ethanol, in which they were stored until DNA extraction for genotyping and sequencing. Total DNA was extracted by the salting-out method [44] and resuspended in 50 µl of 10 mM Tris-HCl–1 mM EDTA, pH 7.8.

### Microsatellite Genotyping and Statistical Analysis

We used 10 microsatellite loci to genotype a subsample of 610 *S. titanus* individuals. The technical protocol for nine of these loci (*Sti6, Sti15, Sti34, Sti36, Sti38, Sti46, Sti64, Sti68-08* and *Sti80*) has been described elsewhere [45]. An additional newly developed locus, Sti31-07, was also used, according to the technical details provided in Table 2. PCR products were sized on a Beckman Coulter Ceq8000 automated sequencer, using the manufacturer’s fragment detection chemistry. Seven of the French sites (Couhins, Seyches, Monein, Margaux, Boutiers, Assas and Valréas) studied here with the 10 microsatellite loci were also investigated with seven of these markers in a previous study by Papura *et al*. [46].

**Table 2:** Technical details concerning the Sti31-07 microsatellite locus.

Locus	Primer sequence (5′–3′)	Repeat motif	T_a_ (°C)	Size range of alleles (bp)	No.of alleles	*H*_O_	*H*_E_	GenBank Accession no.
Sti31	F : AGTTCCCACAAGTGACCGTA R : TTTCCACACATTTTGTCTTCAA	(GA)_12_GT(GA)_2_	50	158–168	5	0.375	0.723	JN675926

T_a_, annealing temperature; *H*_O_, observed heterozygosity, and *H*_E_, expected heterozygosity were calculated for 229 individuals of *S. titanus* sampled over a large geographic scale, from European (N = 125) and American vineyards (N = 104).

Genetic variation within populations was quantified by estimating allelic frequencies, the mean number of alleles per locus (A) and unbiased heterozygosity, (*H*) [47], which were calculated with GENEPOP 3.3 [48]. The interpretation of interpopulation differences may be complicated if sample sizes are unequal [49]. We therefore used FSTAT 2.9.3.2. [50] to calculate allelic richness in NE American and Western European populations. We used the nonparametric Wilcoxon rank sum test to compare mean values for allelic richness and expected heterozygosity (*H_E_*) between the NE American and European populations, for each of the 10 microsatellite loci studied. These statistical analyses were carried out with R 2.8.1 [51].

Deviations from Hardy-Weinberg equilibrium and unbiased estimates of *F*_IS_ [52] were calculated with FSTAT 2.9.3.2. [50]. Null allele frequencies were estimated for each locus and population with FreeNA [53] (available at http://www1.montpellier.inra.fr/URLB/), according to the expectation maximization (EM) algorithm of Dempster *et al*. [54]. Exact tests of global linkage disequilibrium between pairs of loci were carried out with GENEPOP 3.3 [48].

Population structure was assessed by calculating the pairwise *F*_ST_ between populations, with excluding null alleles (ENA) correction, implemented in FreeNA, to correct for the positive bias induced by the presence of null alleles [54]. The 95% confidence intervals for corrected pairwise *F*_ST_ values were obtained by bootstrapping 2000 times over loci. As described by Rousset [55], isolation by distance (IbD) was tested by determining whether genetic distance (pairwise *F*_ST_/(1-*F*_ST_)) and the logarithm of geographic distance for pairs of populations were correlated, in Mantel test using GENEPOP. We used 1500 permutations to assess significance.

Neighbor-joining trees were constructed for populations [56] with POPULATIONS 1.2.30 (O Langella, http://ppa.launchpad.net/olivier-langella/) and the Cavalli-Sforza and Edwards chord distance. Bootstrap values were calculated by resampling loci and are given as a percentage of 2000 replicates.

The Bayesian clustering approach implemented in STRUCTURE version 2.3.3 [57] was used to infer the number of clusters (K) in the dataset for all NE American and European *S. titanus* populations considered together and for American and European populations considered separately. The software was run with the admixture option, allowing for some mixed ancestry within individuals. Ten independent runs were carried out for each value of K (K = 1–8), with a burn-in period of 100,000 iterations and 500,000 replications. We used the method described by Evanno et al. [58] to determine the most likely number of clusters.

Finally, we used the method of Rannala and Mountain [59] to assign the European individuals to either NE American or European populations. The most probable source for each European population was identified by calculating the mean likelihood of the multilocus assignment of an individual from introduced population (*i*) to each possible American or European source population (*s*) by GENECLASS2 [60]. We first assessed the accuracy with which this assignment method distinguished between population sources, by assigning NE American individuals to NE American populations.

### Mitochondrial COII Sequence Data and Statistical Analyses

We analyzed a 623 bp mtDNA fragment containing a portion of the cytochrome oxidase subunit II and tRNAleu genes (referred to hereafter as tRNA^LEU^-COII), previously studied by Moran *et al*. [61] for Cicadellinae, with the insect universal PCR primers mtd 13 (5′-AAT ATG GCA GAT TAG TGC A-3′) and mtd 18 (5′ CCA CAA ATT TCT GAA CAT TGA CCA 3′). Primers were synthesized with an additional forward/reverse M13/pUC sequence at the 5′ end of the mtd13 and mtd18 primers, respectively. PCR was performed in a final volume of 25 µl containing 0.75 U *Taq* polymerase and 1 x buffer (Invitrogen), 0.2 µM of each primer, 0.15 mM of each dNTP, 2 mM MgCl_2_, and 2.5 µg bovine serum albumin. The mixture was heated at 94°C for 4 minutes and then subjected to 30 cycles of 45 s at 94°C, 30 s at 50°C, 1 min at 72°C and a final extension step of 10 min at 72°C.

PCR products were purified with the Qiaquick PCR purification kit (Qiagen). Sequencing reactions were performed with the SequiTherm EXCEL™ II DNA Sequencing Kit (Epicentre Biotechnologies), with the M13/pUC forward and reverse labeled primers. Sequencing was carried out with a 4000 L automatic DNA sequencer (Li-Cor) and 6% Long Ranger (TEBU) gels.

MtDNA data analyses were performed for all populations for which at least three individuals had been sequenced: 22 European and six American populations (Table 1). The haplotype network was constructed with TCS 1.21 [62]. Diversity and differentiation were analyzed with PERMUT [63], [64], which can be used to determine whether *G*_ST_ is significantly smaller than *N*_ST_, which takes into account the genetic distances between haplotypes. Diversity indices and pairwise differentiation were assessed with ARLEQUIN 3.11 [65]. As for the microsatellite data, IbD was investigated by testing the correlation between genetic differentiation (expressed as *F*_ST_/(1-*F*_ST_)) and the logarithm of geographic distance for all pairs of populations in a Mantel test [55], [66].

## Results

### Microsatellite Data

The 10 microsatellite loci displayed various degrees of polymorphism, each having five (Sti15 and Sti80) to 39 (Sti36) alleles, with a mean of 21 alleles per locus for all samples. In total, 217 alleles observed, 115 of which were found in both NE American and European individuals, 84 of which were found only in NE American populations and 18 of which were specific to Europe. Observed heterozygosities (*H*_O_) ranged from 0.398 to 0.581 for NE American populations and from 0.369 to 0.535 for European populations. Expected heterozygosities (*H*_E_) were between 0.692 and 0.771 for NE American populations and from 0.543 to 0.675 for European populations (Table 3). Genetic diversity and allelic richness were significantly higher in NE America than in Europe (*H*_E_ was calculated from each of 10 loci and compared between the two continents; Wilcoxon rank sum test *P* = 0.012 and *P* = 0.009, respectively).

**Table 3:** Summary of genetic variation at 10 microsatellite loci scored for 10 American and 14 European *S. titanus* populations.

Populations	N	AR	*H*_O_	*H*_E_	F_IS_	*F*_ST_
					multiloci	
***Western Europe***						
Boutiers	32	3.238	0.418	0.642	0.365	
Couhins	30	3.261	0.391	0.665	0.431	
Seyches	12	3.517	0.535	0.674	0.257	
Monein	30	3.315	0.446	0.658	0.338	
Assas	30	3.356	0.511	0.675	0.260	
Mudaisson	25	3.219	0.527	0.641	0.207	
Valréas	30	3.121	0.393	0.647	0.409	
Casamozza	25	3.044	0.423	0.621	0.339	
Anierès	24	3.192	0.439	0.654	0.355	
Castelrotto	24	2.678	0.369	0.543	0.345	
Pedrinate	24	3.149	0.508	0.642	0.229	
Novaledo	30	3.070	0.468	0.628	0.277	
Arco	28	3.174	0.422	0.633	0.355	
Tramonti	33	3.045	0.439	0.606	0.317	
Total Europe	377	3.170	0.449	0.638	0.320	0.046
(Mean±SD)		(±0.191)	(±0.053)	(±0.033)	(±0.066)	(±0.021)
***North-Eastern America***						
Niagara 1	22	3.987	0.581	0.771	0.269	
Niagara 2	26	3.859	0.546	0.740	0.281	
Geneva 1	15	3.583	0.462	0.692	0.365	
Geneva 2	19	3.831	0.533	0.750	0.316	
Geneva 3	30	3.758	0.451	0.729	0.397	
Cornell	45	3.581	0.542	0.752	0.290	
Dresden	24	3.796	0.453	0.695	0.367	
Hither Hills 1	26	3.874	0.476	0.738	0.373	
Hither Hills 2	15	3.841	0.398	0.735	0.490	
Rio Grande	11	3.794	0.579	0.712	0.233	
Total NE America	233	3.790	0.502	0.731	0.338	0.042
(Mean±SD)		(±0.125)	(±0.062)	(±0.025)	(±0.075)	(±0.021)

N, sample size; A, mean number of AN,N, number of *S. titanus* individuals sampled from each site; AR, allelic richness; *H*_E_ and *H*_O_, expected and observed heterozygosities; *F*_IS_, estimates of *F*_IS_ values; *F*_ST_, index of genetic differentiation [52].

Significant heterozygote deficiencies and significant positive *F*_IS_ values were observed in all populations (Table 3). Moreover, the estimated proportions of null alleles per locus and per population were systematically above 10% for three microsatellite loci: *Sti36, Sti38* and *Sti68*. We thus repeated the data analyses without these markers. After these three loci had been removed, the dataset still showed significant heterozygote deficiencies in all NE American populations and in seven of the 14 European populations (Boutiers, Casamozza, Couhins, Seyches, Valréas, Anierès and Pedrinate).

Significant global linkage disequilibrium was found only in three of the 1080 pairwise tests carried out, for Sti15 & Sti80 (Hither Hills-2), Sti6 & Sti64 (Assas) and Sti15 & Sti46 (Anierès). The 10 microsatellite markers were thus considered independent.

### Population Genetic Structure

Most pairwise sample comparisons with ENA correction for the presence of null alleles showed significant genetic differentiation after correction for multiple comparisons (*P*<0.05): 130 of the 137 pairwise *F*_ST_ values were significantly different from zero (Table 4). Within NE America, pairwise *F*_ST_ values ranged from 0 (between Geneva-2 and Geneva-3, which are 4 km apart) to 0.082 (Hither Hills-1 and Geneva-1, 286 km apart), with a mean pairwise *F*_ST_ value of 0.042 (SD = 0.021, Table 5). Within Europe, *F*_ST_ values ranged from 0.001 (between Boutiers and Seyches, 178 km apart) to 0.109 (Castelrotto and Seyches, 689 km apart) and the mean pairwise *F*_ST_ was 0.046 (SD = 0.021, Table 4). Maximal *F*_ST_ values were obtained for comparisons of NE American and European populations (mean pairwise *F*_ST_ values = 0.125, SD = 0.030).

**Table 4:** Pairwise estimates of *F*_ST_ and mean individual assignment likelihood (L_i→s_) for NE American and European populations.

North-Eastern America											Western Europe													
	Hither1	Hither2	Niagara1	Niagara2	Geneva1	Geneva2	Geneva3	Dresden	Cornell	RioGrande	Assas	Boutiers	Casamozza	Couhins	Monein	Mudaisson	Seyches	Valréas	Arco	Novaledo	Tramonti	Anierès	Castelrotto	Pedrinate
Hither1	_	_	_	_	_	_	_	_	_	_	_	_	_	_	_	_	_	_	_	_	_	_	_	_
Hither2	0.008	_	_	_	_	_	_	_	_	_	_	_	_	_	_	_	_	_	_	_	_	_	_	_
Niagara1	0.032	0.026	_		_	_	_	_	_	_	_	_	_	_	_	_	_	_	_	_	_	_	_	_
Niagara2	0.047	0.046	0.031	_	_	_	_	_	_	_	_	_	_	_	_	_	_	_	_	_	_	_	_	_
Geneva1	0.081	0.060	0.0612	0.063	_	_	_	_	_	_	_	_	_	_	_	_	_	_	_	_	_	_	_	_
Geneva2	0.059	0.042	0.034	0.050	0.007	_	_	_	_	_	_	_	_	_	_	_	_	_	_	_	_	_	_	_
Geneva3	0.060	0.051	0.050	0.052	0.016	0.000	_	_	_	_	_	_	_	_	_	_	_	_	_	_	_	_	_	_
Dresden	0.077	0.056	0.054	0.065	0.027	0.004	0.000	_	_	_	_	_	_	_	_	_	_	_	_	_	_	_	_	_
Cornell	0.050	0.052	0.027	0.047	0.048	0.024	0.021	0.024	_	_	_	_	_	_	_	_	_	_	_	_	_	_	_	_
RioGrande	0.024	0.033	0.025	0.042	0.081	0.057	0.057	0.081	0.035	_	_	_	_	_	_	_	_	_	_	_	_	_	_	_
Assas	0.066	0.082	0.064	0.089	0.120	0.105	0.107	0.120	0.082	0.067	_	0.069	0.041	0.043	0.021	0.019	0.053	0.053	0.055	0.059	0.023	0.012	0.076	0.038
	(**12.359**)	(13.564)	(14.952)	(15.622)	(15.918)	(16.415)	(15.203)	(16.347)	(15.920)	(13.753)		(19.325)	(17.477)	(17.010)	(17.805)	(17.784)	(15.824)	(17.763)	(19.031)	(17.808)	(17.127)	(16.625)	(19.041)	(16.586)
Boutiers	0.133	0.126	0.101	0.147	0.130	0.125	0.134	0.152	0.127	0.105	0.069	_	0.051	0.020	0.041	0.046	0.001	0.030	0.070	0.058	0.043	0.052	0.087	0.043
	(15.094)	(**13.917**)	(15.702)	(18.412)	(16.625)	(18.271)	(16.613)	(16.655)	(19.024)	(15.381)	(17.372)		(18.422)	(16.876)	(18.416)	(17.797)	(14.719)	(16.222)	(19.080)	(17.765)	(16.549)	(16.690)	(18.640)	(15.593)
Casamozza	0.127	0.144	0.096	0.157	0.161	0.136	0.151	0.161	0.113	0.121	0.041	0.051	_	0.053	0.045	0.039	0.036	0.063	0.037	0.040	0.054	0.034	0.088	0.021
	(**13.543**)	(14.918)	(15.674)	(18.858)	(17.745)	(17.529)	(17.376)	(17.750)	(17.865)	(14.561)	(17.610)	(19.552)		(18.579)	(18.498)	(17.807)	(15.130)	(17.640)	(18.234)	(17.392)	(17.852)	(15.916)	(19.036)	(15.493)
Couhins	0.109	0.100	0.081	0.121	0.127	0.112	0.115	0.132	0.105	0.088	0.043	0.020	0.053	_	0.038	0.039	0.009	0.041	0.059	0.056	0.042	0.035	0.079	0.037
	(14.359)	(14.654)	(16.349)	(16.881)	(16.995)	(17.848)	(16.247)	(16.735)	(17.964)	(**14.085**)	(17.028)	(18.384)	(18.204)		(18.655)	(17.614)	(15.594)	(16.829)	(20.666)	(17.929)	(16.924)	(15.878)	(19.112)	(17.463)
Monein	0.082	0.083	0.067	0.096	0.122	0.116	0.123	0.133	0.092	0.077	0.021	0.041	0.045	0.038	_	0.009	0.043	0.043	0.046	0.064	0.013	0.027	0.048	0.033
	(14.047)	(**13.747**)	(16.096)	(17.953)	(18.3159)	(18.612)	(16.929)	(17.212)	(18.386)	(15.277)	(16.762)	(18.266)	(17.118)	(17.384)		(16.917)	(15.503)	(17.316)	(18.287)	(17.202)	(16.424)	(15.672)	(18.527)	(15.595)
Mudaisson	0.112)	0.115	0.092	0.130	0.160	0.149	0.154	0.163	0.120	0.102	0.019	0.046	0.039	0.039	0.009	_	0.050	0.051	0.033	0.059	0.015	0.022	0.051	0.023
	(**13.006**	(13.588)	(14.250)	(16.719)	(17.644)	(17.741)	(15.204)	(15.671)	(16.527)	(14.174)	(16.746)	(17.945)	(16.455)	(17.188)	(16.783)		(15.194)	(16.817)	(17.186)	(16.291)	(16.089)	(15.537)	(17.719)	(13.657)
Seyches	0.108)	0.109	0.077	0.131	0.104	0.093	0.101	0.125	0.100	0.089	0.053	0.001	0.036	0.009	0.043	0.050	_	0.013	0.066	0.062	0.049	0.034	0.109	0.027
	(15.830	(**14.564**)	(16.853)	(19.590)	(17.428)	(18.981)	(18.742)	(17.01975)	(19.300)	(15.828)	(17.520)	(17.972)	(18.020)	(17.333)	(18.460)	(17.541)		(15.937)	(19.637)	(18.039)	(17.156)	(16.109)	(18.468)	(15.876)
Valréas	0.109	0.108	0.091	0.120	0.104	0.105	0.112	0.125	0.105	0.091	0.053	0.030	0.063)	0.041	0.043	0.051	0.013	_	0.072	0.064	0.047	0.038	0.100	0.036
	(15.698)	(**13.887**)	(17,357)	(18.825)	(16.600)	(17.279)	(16.885)	(17.127)	(18.838)	(16.506)	(18.031)	(17.582)	(19.018	(17.101)	(18.765)	(17.723)	(15.401)		(19.797)	(17.770)	(16.605)	(16.382)	(19.515)	(16.943)
Arco	0.131	0.146	0.109	0.157	0.186	0.172)	0.186	0.199	0.144	0.141	0.055	0.070	0.037	0.059	0.046	0.033	0.066	0.072	_	0.064	0.052	0.056	0.067	0.030
	(**15.054**)	(15.877)	(15.694)	(17.897)	(18.792)	(18.774	(18.535)	(17.682)	(18.014)	(15.203)	(17.909)	(19.170)	(17.307)	(18.472)	(18.118)	(17.483)	(15.710)	(18.686)		(17.192)	(17.221)	(16.631)	(19.991)	(15.069)
Novaledo	0.139	0.141	0.104	0.165	0.172	0.149	0.159	0.163	0.128	0.122	0.059	0.058	0.040	0.056	0.064	0.059	0.062	0.064	0.052	_	0.085	0.049	0.098	0.047
	(14.746)	(**14.654**)	(16.808)	(17.766)	(17.558)	(17.601)	(16.982)	(18.497)	(17.847)	(15.341)	(18.057)	(18.730)	(17.413)	(17.831)	(18.702)	(17.527)	(15.852)	(18.437)	(18.484)		(17.046)	(16.737)	(18.753)	(16.744)
Tramonti	0.120	0.125	0.099	0.127	0.145	0.146	0.148	0.162	0.126	0.107	0.023	0.043	0.054	0.042	0.013	0.015	0.049	0.047	0.052	0.085	_	0.036	0.041	0.035
	(12.128)	(13.306)	(**12.068**)	(15.035)	(13.165)	(13.383)	(14.001)	(13.688)	(14.206)	(14.137)	(15.961)	(17.743)	(17.203)	(17.931)	(16.932)	(18.229)	(14.710)	(13.597)	(17.456)	(16.862)		(15.120)	(17.532)	(14.517)
Anierès	0.087	0.090	0.067	0.108	0.131	0.114	0.116	0.127	0.097	0.079	0.012	0.052	0.034	0.035	0.027	0.022	0.034	0.038	0.056	0.049	0.036	_	0.077	0.020
	(**11.987**)	(13.184)	(14.839)	(16.814)	(16.018)	(16.909)	(16.845)	(16.426)	(17.442)	(12.125)	(15.702)	(17.037)	(15.688)	(16.811)	(16.606)	(15.482)	(14.390)	(15.636)	(17.374)	(15.686)	(14.400)		(16.779)	(14.843)
Castelrotto	0.150	0.158	0.131	0.163	0.192	0.194	0.198	0.209	0.165	0.151	0.076	0.087	0.088	0.079	0.048	0.051	0.109	0.100	0.067	0.098	0.041	0.077	_	0.067
	(**11.951**)	(13.867)	(14.052)	(16.282)	(17.475)	(18.019)	(16.441)	(15.808)	(17.519)	(12.625)	(14.360)	(15.647)	(13.765)	(15.860)	(14.755)	(15.003)	(12.969)	(13.588)	(14.925)	(15.216)	(14.147)	(12.370)		(12.237)
Pedrinate	0.115	0.121	0.096	0.153	0.160	0.144	0.155	0.168	0.127	0.122	0.038	0.043	0.021	0.037	0.033	0.023	0.027	0.036	0.030	0.047	0.035	0.020	0.067	_
	(**12.872**)	(13.624)	(16.102)	(18.390)	(16.933)	(17.269)	(17.101)	(17.609)	(17.782)	(14.216)	(18.030)	(19.030)	(17.280)	(18.428)	(18.290)	(17.270)	(15.203)	(17.748)	(18.551)	(17.350)	(17.491)	(16.182)	(17.875)	

All pairwise *F*_ST_ are significant (p<0.05), except when followed by (NS) (non-significant) The pairwise estimate values of *F*_ST_ are indicated on the lower half of the matrix and L_i→s_ values expressed on a –log scale are indicated on the upper half of the matrix for the European population only. For each European population, the maximum of L_i→s_ are indicated in bold typeface. * indicated the second most likely source of two European populations (Arco and Castelrotto) which were firstly assigned to.

The pairwise *F*_ST_ matrix was obtained for all microsatellite loci, by applying ENA correction for null alleles with FreeNA. Most *F*_ST_ pairwise differentiations were significant after correction for multiple comparisons, with the exception of those between Hither Hills1 and Hither Hills2, Geneva1 and Geneva2, Geneva2 and Geneva3, Geneva3 and Dresden, Seyches and Boutiers, Seyches and Couhins, Monhein and Mudaisson; L_i→s_ values, expressed on a –log scale, obtained with the seven loci having no more than 10% null alleles, are indicated in parentheses for the European samples only. For each European sample, the minimum value of –log(L_i→s_) is indicated in bold typeface and underlined.

**Table 5:** Comparative overall population genetic structure of the NE American and European populations.

Genomic class	Parameter		North-Eastern America		Western Europe
Nuclear	*H*_E_		0.731 (+/−0.025)		0.638 (+/−0.033)
	A		9.2 (+/−1.3)		6.2 (+/−0.8)
	*G*_ST_		0.045		0.047
	*F*_ST_		0.042 (+/−0.021)		0.046 (+/−0.021)
	IbD		YES (r = 0.202, P = 0.020)		NO (r = 0.093, P = 0.783)
Mitochondrial	*H*		0.583 (+/−0.072)		0.203 (+/−0.042)
	π	0.0046 (+/−0.0027)		0.0013 (+/−0.0001)	
	*G*_ST_	0.056		0.012	
	*N*_ST_		0.077*		0.018 (NS)
	IbD		YES (*r* = 0.87, *P* = 0.003)		NO (*r* = −0.09, *P* = 0.783)

A, mean number of alleles per locus, *H*_E_ expected heterozygosity. Indices of genetic differentiation: *F*_ST_, *G*_ST_ and *N*_ST_; IbD, isolation by distance; π = mean nucleotide diversity over all loci; +/−, standard deviation values; * indicates that *N*_ST_ is significantly higher than *G*_ST_ in NE America, whereas the difference between *N*_ST_ and *G*_ST_ was not significant in Europe.

Genetic distances (*F*_ST_/1-*F*_ST_) between pairs of populations were weakly but significantly correlated with geographic distance for NE American sample sites (Mantel test, *r* = 0.202, *P* = 0.020), whereas no pattern of IbD was detected for European populations (*r* = 0.093, *P* = 0.783).

**Figure 2:**
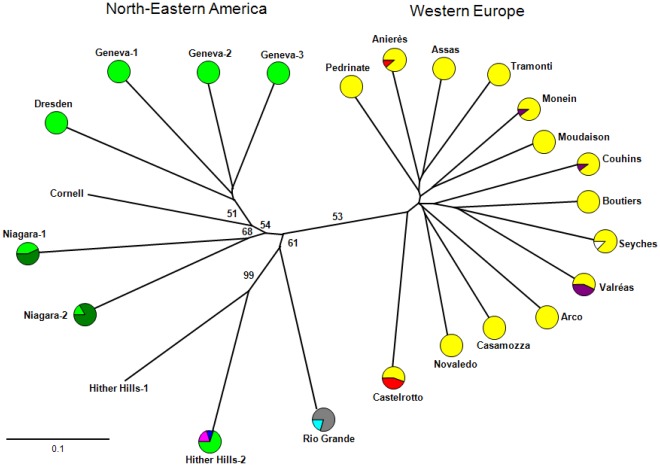
Neighbor-joining tree based on Cavalli-Sforza and Edwards’ chord distances between populations derived from the allelic frequencies of 10 microsatellite loci. Bootstrap values (2000 replications, with resampling) are indicated as percentages. The frequencies of mitochondrial haplotypes are shown in the pie charts for each population for which at least three individuals were sequenced. The size of the circles is not proportional to the number of individuals sampled per population; the colour codes are as in Figure 1.

In the neighbor-joining tree (built with Cavalli-Sforza and Edward’s chord distances for 10 microsatellite loci), the *S. titanus* populations clearly clustered into two groups: 1) the European populations, which grouped together with poor clade resolution and 2) the American populations, which clustered into four subgroups (with more than 50% bootstrap support): the first subgroup consisted of the Geneva-1, Geneva-2, Geneva-3, Cornell and Dresden populations, the second subgroup consisted of the Niagara-1 and Niagara-2 populations, the third subgroup consisted of the Hither Hills-1 and Hither Hills-2 populations and the last subgroup consisted of the Rio Grande population alone (Figure 2). This topology reflects the geographic location of the American populations. Limitation of the analysis of genetic differentiation to the seven loci (*Sti6, Sti15, Sti31-07, Sti34, Sti46, Sti64* and *Sti80*) with no more than 10% null alleles had no significant effect on the results obtained (data not shown).

**Figure 3:**
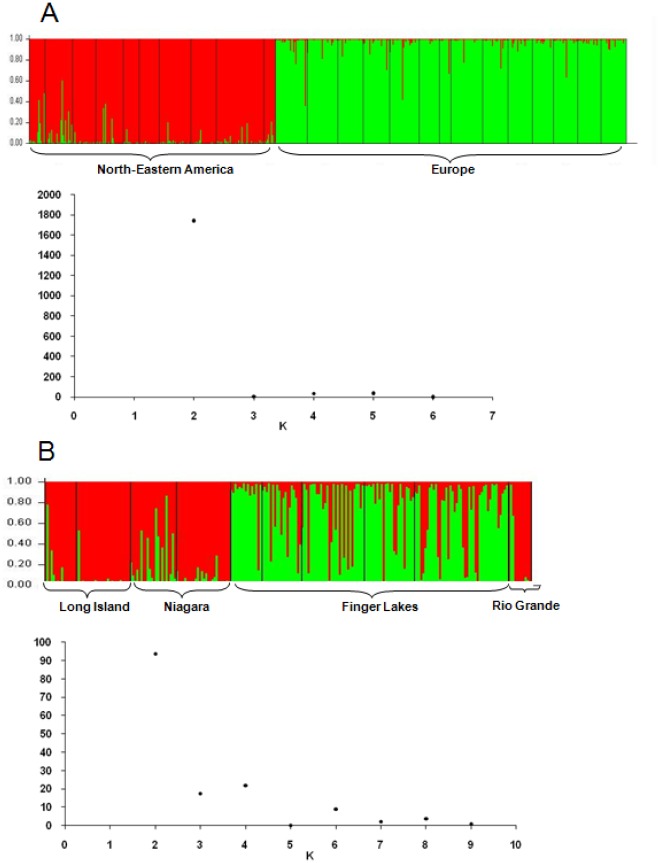
Estimation of population genetic structure by Bayesian analysis: A with K = 2 clusters, for the analysis of all NE American and European samples and B with K = 2 clusters for the analysis of NE American samples alone.

We used a Bayesian clustering method to detect possible cryptic substructure in the whole dataset (NE America and Europe). The ▵K statistic [58] indicated that NE American and European individuals were separated in two distinct groups (the highest ▵K value was obtained with K = 2, Figure 3.A). When the NE American samples were analyzed separately, two groups of individuals were defined: the first including individuals from the Long Island, Niagara Peninsula and Rio Grande regions and the second grouping together individuals from Finger Lake (Figure 3.B). When the European *S. titanus* samples were analyzed separately, no particular structure could be identified, consistent with the results of neighbor-joining tree analysis (Figure 2). Restriction of the Bayesian clustering analysis to the seven loci with no more than 10% null alleles had no effect on the results obtained (data not shown).

### Identification of the Most Plausible Source of the European Populations

Null alleles cause a bias in assignment tests, decreasing the power to assign individuals to their source populations correctly [67]. We therefore limited this analysis to the seven loci with less than 10% null alleles (*Sti6, Sti15, Sti31-07, Sti34, Sti46, Sti64* and *Sti80*). We first checked the efficacy of this technique by assigning NE American individuals to NE American populations: 96% of the individuals were assigned to the correct NE American population (*P*<0.05). By contrast, control assignment tests placed European leafhoppers in their correct European populations for only 76.3% of the individuals, consistent with the low level of population differentiation observed in Europe. The most probable source of each European population was determined by analyzing the mean likelihood of assigning an individual from sample *i* to sample *s* (L_i→s_ values expressed on a –log scale). The deduced most probable source for each European site was identified as the sample with the lowest –log(L_i→s_) values (Table 4). These criteria identified the NE American site on the Atlantic coast at which the host plant *V. aestivalis* occurs (Hither Hills) as the most plausible source for 12 of the 14 European populations (Table 4). For one population from western France (Couhins) the Rio Grande population (*V. aestivalis* grows at this site) was identified as the most probable source and for one population from Southern Italy (Tramonti), a Niagara Peninsula population (where *V. vinifera* grows) was identified as the most probable source. No European population was found to have a most probable source population in the Finger Lakes region (where *V. riparia* grows). Differences in –log(L_i→s_) between the various putative sources were small. We were unable to assess the significance of these differences because there is currently no statistical procedure available for doing so [68] (see Ciosi et al. 2008 for a discussion on this point).

### Mitochondrial DNA

tRNA^LEU^-COII MtDNA fragments from one to 11 individuals from 30 populations were successfully sequenced (N = 214, Table 1). Ten haplotypes were identified in eight American and 22 European populations. The MtDNA sequences of each of these haplotypes are available from GenBank under accession numbers JN547229-JN547238. Four haplotypes were found in Europe and six in North America, but none was common to both regions. Moreover, the European haplotypes did not form a differentiated lineage (Fig. 1c). The haplotypes found in NE America and in Europe differed by one to 10 mutations.

In Europe, the maximum divergence between the haplotypes found in different populations was six mutation steps, whereas that between the haplotypes within a population was five mutation steps. One major haplotype (i) was widely distributed throughout Europe and was present in each of the populations sampled, whereas two rarer haplotypes (g and j) were found across the South of France (both the south-western and south-eastern regions) but were apparently absent from Switzerland and Italy. One haplotype (h) was restricted to Switzerland (Fig. 1).

In North America, the most distant haplotypes found in different populations were separated by 11 mutation steps, whereas haplotypes present in populations from the same site differed by no more than seven mutations. Haplotype distribution was independent of host plant. Two closely related haplotypes were found in the Niagara Peninsula and Finger Lakes regions. The major haplotype (b) was also present in the Long Island region, on the Atlantic Coast, where the two other haplotypes (e and f) genetically close to those in Europe were found. Haplotype f occupies a central position in the unrooted network, but was found in only two American individuals. The southernmost population from Rio Grande had only private haplotypes in our sampling (c and d, Fig. 1).

Genetic and nucleotide diversities were higher in America than in Europe (Table 5). The level of differentiation between populations was also higher in NE America (*G*_ST_ = 0.056) than in Europe (*G*_ST_ = 0.012). Moreover, *N*_ST_ reached 0.077 and was significantly higher than *G*_ST_ in America, whereas the difference between *N*_ST_ and *G*_ST_ was not significant in Europe (*N*_ST_ = 0.018, Table 5).

A significant pattern of IbD was found in American populations (Mantel test *r* = 0.87, *P = *0.003), whereas no such isolation was detected in Europe (*r* = −0.09, *P* = 0.783, Table 5). These results are consistent with our findings for microsatellite data.

## Discussion

In this study, we combined analyses of nuclear and mitochondrial markers to trace the routes by which *S. titanus*, an invasive leafhopper, was introduced into Europe. One of the advantages of looking for concordance across independent loci within a species is that congruent patterns are likely to reflect common historical events, reducing the sampling variance of the lineage sorting process [69]. Both nuclear and mtDNA markers classified the populations into two distinct groups: the NE American *S. titanus* populations displayed high levels of variability and geographic structure, whereas, as expected, the European populations in the colonized region displayed lower levels of genetic diversity and no IbD. We obtained no evidence to suggest that *S. titanus* had been introduced into Europe more than once. For most of the samples from the introduced geographic range, populations from the Atlantic Coast winegrowing region in which *V. aestivalis* occurs were considered the most likely source.

### Heterozygote Deficiency

Significant heterozygote deficiency was observed in both native and introduced populations. For three out of the ten microsatellite loci studied, technical biases may have been responsible for the observed homozygote excess, these loci being estimated to have more than 10% null alleles. However, even after the elimination of these loci from the analysis, significant deviations from Hardy-Weinberg equilibrium remained. The heterozygote deficiency may be due to biological factors or sampling biases, such as inbreeding or the Wahlund effect (sampling of individuals actually originating from different genetic pools). Both these explanations are possible for *S. titanus*. Nymphal aggregation in vineyards has been reported for this species [70], potentially resulting in consanguineous mating at a local scale and accounting for the higher degree of relatedness and homozygosity of the individuals sampled within a population. Alternatively, the homozygote excess could be accounted for by the Wahlund effect, as the insects sampled in a given vineyard may actually have come from different populations in the surrounding woodland vegetation. This hypothesis is particularly plausible in native habitats, in which *S. titanus* also grows on uncultivated host plants outside the vineyards [20], [22].

### Native Populations: Genetic Variability and Phylogeographic Structure

A clustering analysis of nuclear markers showed that the NE American populations formed a well defined group that was differentiated from the introduced European *S. titanus* populations. Furthermore, within this native group, both mitochondrial and nuclear markers showed there to be a significant genetic structure and IbD. The level of heterozygosity was high and almost all the alleles observed (92%) were found in NE America, consistent with the location of this region within the native range of *S. titanus*. The American populations were geographically structured. This phylogeographic pattern was confirmed by mitochondrial data, as the differentiation index *N*_ST_ (taking into account similarity between haplotypes) was significantly higher than *G*_ST_ (based solely on haplotype identity). The concordance of mitochondrial and nuclear results suggests that the main patterns of dispersion are similar for males and females in *S. titanus*.

In three of the four vine-growing areas sampled in NE America, a single cultivated *Vitis* sp. predominated. It is therefore difficult to disentangle the effects of host plant and geographic location. However, in the Niagara region, where insects were sampled from two different host plants (*Vitis vinifera* and *V. labrusca*), no differentiation as a function of host plant was found. We therefore hypothesize that geographic location may be the main factor underlying the differentiation between populations in the native area.

### European Populations: Genetic Diversity and Structure

Levels of genetic diversity were significantly lower in European than in American populations, in analyses of both the mitochondrial and nuclear genomes. The founder effect resulting from the recent introduction of this insect in Europe [71] and the expansion process following its introduction, with sequential bottlenecks, may account for the observed decrease in genetic diversity [72], [73], [74], [75]. Nevertheless, European populations had an allelic diversity about 65% that in the native range and displayed 87% heterozygosity. As pointed out in several reviews, large losses of variation are actually rare in invaders, with many species resolving this paradox by somehow maintaining genetic diversity during colonization [76], [77]. Our results are consistent with the general patterns outlined in a recent review of findings for 80 introduced species of animals, plants and fungi, in which nuclear molecular diversity was compared between introduced and source populations. This review showed a significant 32.7% overall loss of allelic diversity and a significant mean loss of 22.6% heterozygosity in introduced populations with respect to source populations [78]. These figures are very similar to those obtained here.

By contrast to our findings for the native range, we found no evidence of genetic structure for *S. titanus* populations in Europe. No significant phylogeographic pattern or IbD was found, with concordant results obtained for nuclear and mitochondrial markers. Weak genetic structure in European populations was also reported by Bertin *et al.* [36] in a study of European populations based on the use of RAPD markers and by Papura *et al.* [46] in studies of some of the French *S. titanus* populations with seven microsatellite loci. The lack of genetic differentiation in European *S. titanus* populations may be indicative of long-distance gene flow (due to insect migration and the transportation by humans of grapevine material carrying eggs). Such long-distance movements of grapevines occurred frequently in the past [79]. The current practice of treating nurseries against *S. titanus* adults (in the framework of compulsory protection regulations) reduces the risk of egg infestations on the material used for plantation, but cannot entirely eliminate this risk.

### Possible Origins of the Introduced Populations

Most of the European individuals were preferentially assigned to source populations from the Atlantic Coast (Long Island region) in which *V. aestivalis* grows. The mitochondrial data were partly consistent with this hypothesis, although the European haplotypes were not found in the American samples. Mitochondrial data showed that NE American populations were phylogeographically structured, the haplotypes found in Long Island being very closely related to the European populations. This suggests that, even if we did not sample the exact source population, it is probably located in the vicinity of the Long Island region. Consequently, of the four NE American wine-growing areas sampled here, Long Island appears to be the most plausible area of origin for the introduced populations of *S. titanus*.

Some recent studies of worldwide introductions have suggested that many widespread invasions may have stemmed from a particularly successful invasive population rather than from the native range. This phenomenon is known as the “invasive bridgehead effect” [80]. One possible scenario for the introduction of *S. titanus* into Europe thus involves the establishment of intermediate invasive populations elsewhere. These intermediate populations would have originated from the native area and served as the immediate source of the invasive European populations. In serial invasion processes of this type, founder events in the intermediate populations may lead to additional genetic bottlenecks. The observed moderate genetic variability reduction in the European populations compared to North American populations of *S. titanus* is consistent with the occurrence of a series of genetic bottlenecks. However, historical data and the known distribution of *S. titanus* worldwide indicate that there are unlikely to be unsampled intermediate populations as this species has only ever been recorded in North America and Europe. The “invasive bridgehead” hypothesis and serial invasion scenarios in general are therefore unlikely for this particular species. Nevertheless, we cannot exclude the possibility that unstudied or even undetected populations from North America (‘ghost populations’) constitute the actual source of the European *S. titanus* populations [68]. Further, larger reservoirs of diversity may exist in North America in the form of populations associated with hosts different from those sampled here. As previously stated, in New York State and Virginia, *S. titanus* also occurs in woodland stands [20], [22]. *S. titanus* populations associated with different natural hosts may therefore be genetically different from those sampled from *Vitis* species and these populations associated with other hosts may be the true source of European populations.

### Colonization Patterns in Europe

The existence in Europe of two main mitochondrial haplotypes – (i), which is common to most of the individuals sampled, and (g), which is present in seven of the 14 French populations – the inference from microsatellite data that there was only one main source for the introduction, and the detection of a single genetic cluster for European populations by both Bayesian analysis and a distance-based Neighbor-joining method suggest that a single introduction event accounts for most, if not all of the individuals in Europe. The high frequency of the rare haplotype (h) in Switzerland may result from genetic drift, which may have favoured a rare haplotype locally that was lost or remained undetected elsewhere. The hypothesis of a second introduction event cannot be completely excluded but is not supported by microsatellite results. More detailed investigation of the distribution of haplotype (h) in Central and Eastern European vineyards is warranted, but it is not currently possible to determine with confidence how many introduction events have occurred. According to historical reports, this insect was introduced into several geographic regions at about the same time: south-western and south-eastern France, and north-western Italy [19], [25], [26]. It seems likely that this pest was first introduced into Europe in the mid-19^th^ century, when large amounts of grapevine material were brought into France from various North American regions for use in breeding programs, initially to combat powdery mildew [81] and then *Phylloxera* [82]. Large numbers of *S. titanus* eggs may therefore have been introduced on several occasions during this period, but probably from the same region of North America. The populations of the first insects introduced may have remained small for a number of years before expanding. Long-distance gene flow within Europe, due to natural migration and the transportation by humans of grapevine canes and grafts carrying eggs, would then account for the genetic homogeneity subsequently observed over a large geographic scale. Future studies based on the use of recently developed methods, such as ABC [83] that take into account stochasticity in calculations of the probabilities of particular introduction scenarios [5], together with broader sampling in the native range of *S. titanus* and more recently colonized countries in Central and Eastern Europe should improve our understanding of the colonization history of this pest species. One of the advantages of ABC analysis is that it can handle unsampled populations. This makes it possible to consider scenarios involving a bridgehead invasive population that has not been sampled.
